# Mollusc-Derived Brominated Indoles for the Selective Inhibition of Cyclooxygenase: A Computational Expedition

**DOI:** 10.3390/molecules26216538

**Published:** 2021-10-29

**Authors:** Md. Mominur Rahman, Md. Junaid, S. M. Zahid Hosen, Mohammad Mostafa, Lei Liu, Kirsten Benkendorff

**Affiliations:** 1 Marine Ecology Research Centre, Faculty of Science and Engineering, Southern Cross University, Lismore, NSW 2480, Australia; m.rahman.23@student.scu.edu.au; 2 Molecular Modeling Drug-design and Discovery Laboratory, Pharmacology Research Division, BCSIR Laboratories Chattogram, Bangladesh Council of Scientific and Industrial Research, Chattogram 4217, Bangladesh; md.junaid@northsouth.edu (M.J.); s.hosen@student.unsw.edu.au (S.M.Z.H.); drmostafainars@bcsir.gov.bd (M.M.); 3 Pancreatic Research Group, South Western Sydney Clinical School, and Ingham Institute for AppliedMedical Research, Faculty of Medicine, University of New South Wales, Sydney, NSW 2052, Australia; 4 Southern Cross Plant Science, Faculty of Science and Engineering, Southern Cross University, Lismore, NSW 2480, Australia; ben.liu@scu.edu.au; 5 National Marine Science Centre, Faculty of Science and Engineering, Southern Cross University, Coffs Harbour, NSW 2450, Australia

**Keywords:** *Dicathais orbita*, inflammation, COX-1/2, molecular docking, molecular dynamics, drug-likeness, pharmacokinetics, toxicokinetics

## Abstract

Inflammation plays an important role in different chronic diseases. Brominated indoles derived from the Australian marine mollusk *Dicathais orbita* (*D. orbita*) are of interest for their anti-inflammatory properties. This study evaluates the binding mechanism and potentiality of several brominated indoles (tyrindoxyl sulfate, tyrindoleninone, 6-bromoisatin, and 6,6′-dibromoindirubin) against inflammatory mediators cyclooxygenases-1/2 (COX-1/2) using molecular docking, followed by molecular dynamics simulation, along with physicochemical, drug-likeness, pharmacokinetic (pk), and toxicokinetic (tk) properties. Molecular docking identified that these indole compounds are anchored, with the main amino acid residues, positioned in the binding pocket of the COX-1/2, required for selective inhibition. Moreover, the molecular dynamics simulation based on root mean square deviation (RMSD), radius of gyration (Rg), solvent accessible surface area (SASA), and root mean square fluctuation (RMSF) analyses showed that these natural brominated molecules transit rapidly to a progressive constant configuration during binding with COX-1/2 and seem to accomplish a consistent dynamic behavior by maintaining conformational stability and compactness. The results were comparable to the Food and Drug Administration (FDA)-approved selective COX inhibitor, aspirin. Furthermore, the free energy of binding for the compounds assessed by molecular mechanics–Poisson–Boltzmann surface area (MM–PBSA) confirmed the binding capacity of indoles towards COX-1/2, with suitable binding energy values except for the polar precursor tyrindoxyl sulfate (with COX-1). The physicochemical and drug-likeness analysis showed zero violations of Lipinski’s rule, and the compounds are predicted to have excellent pharmacokinetic profiles. These indoles are projected to be non-mutagenic and free from hepatotoxicity, with no inhibition of human *ether-a-go–go gene (hERG) I* inhibitors, and the oral acute toxicity LD_50_ in rats is predicted to be similar or lower than aspirin. Overall, this work has identified a plausible mechanism for selective COX inhibition by natural marine indoles as potential therapeutic candidates for the mitigation of inflammation.

## 1. Introduction

Inflammation is an essential part of the immune response system [1] that is initiated by stimuli from pathogens, dust, and oxidative stress, following infection or injury to the tissue [2,3,4]. This biological response includes physiological adaptations that are elicited to eliminate pathogens and initiate wound healing [5]. However, long term chronic or severe acute inflammation can lead to chronic diseases [6], including malignancy [7], neurodegenerative disease [8], rheumatoid arthritis [9], atherosclerosis, liver diseases [10], some lung diseases such as asthma and chronic obstructive pulmonary disease [11,12] as well as bowel disease [13].

Inflammation involves a complex array of functional responses in a cascade of steps [14], involving inflammatory cytokines and other chemical mediators, including prostaglandin [15]. Cyclooxygenase (COX) enzymes are regulatory enzymes that initiate pain, fever, and inflammation through the production of prostaglandin [16]. Also known as prostaglandin-endoperoxide H synthase (PGHS), COX plays a vital role in the conversion of arachidonic acid (AA) into prostanoids [17]. Consequently, COX enzymes are important targets for non-steroidal anti-inflammatory drugs (NSAIDs) [18].

Two related isoforms of COX, formed from multiple genes, have been recognized: COX-1 and COX-2 [19]. COX-1 is mostly considered to be a “housekeeping enzyme” that performs different physiological roles, such as the maintenance of kidney function and the protection of the gastric mucosa. COX-1 is also responsible for the biosynthesis of primary prostanoids, including the regulation of platelet aggregation through thromboxane A2 (TXA2) stimulation [20,21]. By contrast, the gene for COX-2 is a primary response gene with numerous regulatory elements; hence, COX-2 expression can be quickly induced by lipopolysaccharide (LPS) from bacteria, along with cytokines such as tumor necrosis factor-α and interleukin (IL)-1 and the tumor promoter phorbol myristate acetate (PMA) as well as growth factors (GF) [22]. COX-2 is mainly a cytokine-induced isozyme producing prostaglandin I2 (PGI2), and it is ultimately responsible for the initiation and maintenance of the process of inflammation and, consequently, the prevention of platelet aggregation [23,24,25]. Overall, the foremost action of COX-1 is to facilitate the protection of the gastrointestinal tract and modulate platelet and kidney function, while inducible COX-2 is mostly involved in pain and inflammation [26,27,28]. Consequently, selective inhibition of COX-2 is of primary interest for new anti-inflammatory drugs [29], although there is still some degree of interest in COX-1 inhibition [20]. The involvement of COX-1 in inflammation and cancer has been firmly recognized [30].

From ancient times, mollusks have been used to treat inflammatory diseases [31]. Recently, heterocyclic compounds from the black clam *Villorita cyprinoides* were investigated using the in silico approach for COX inhibition [32]. A significant docking score and binding energy, along with good interaction with amino acid residues in the active site of COX-2, demonstrated the potentiality of this mollusk for COX-2 inhibition. The Muricidae family of shelled caenogastropods is known to contain bioactive heterocyclic compounds [33]. Bioassay-guided fractionation of anti-inflammatory extracts from the hypobranchial glands of the Australian muricid *D. orbita* revealed 6-bromoisatin as a potent inhibitor of nitric oxide (NO), tumor necrosis factor-alpha (TNFα), and prostaglandin in vitro [34]. Subsequently, an in vivo model for acute lung inflammation in mice confirmed the anti-inflammatory activity of 6-bromoisatin and the mollusk hypobranchial gland extract [35]. Some related secondary metabolites from this mollusk, such as tyrindoleninone and 6,6′dibromoindirubin, have also been observed to have anti-cancer and anti-inflammatory properties [34,36,37]. Nevertheless, to date, there appears to have been no studies that have investigated whether these molluscan brominated indole derivatives can specifically target COX isoforms.

**Figure 1:**
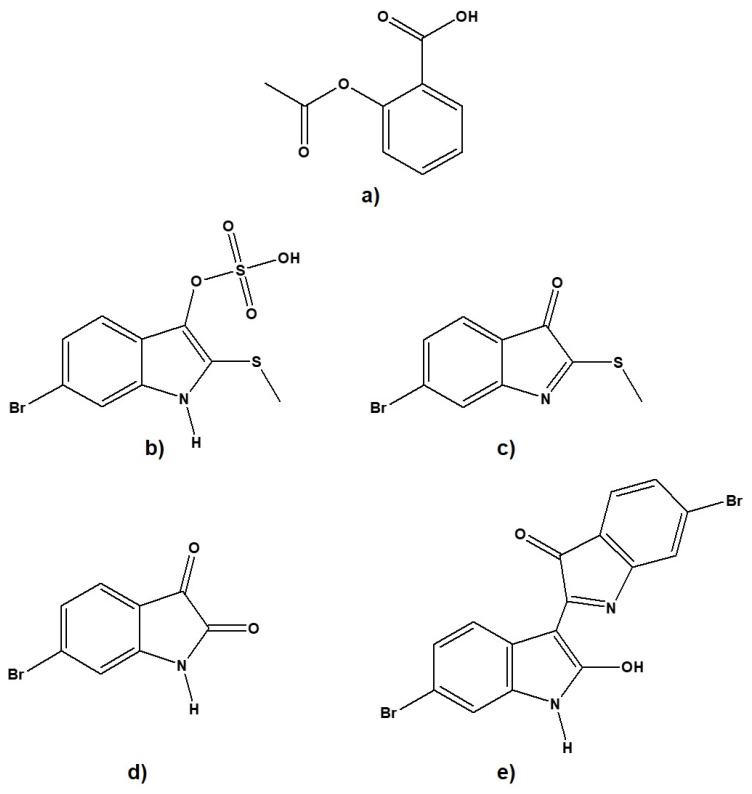
The 2D structure of the ligands used in this study. (**a**) aspirin, (**b**) tyrindoxyl sulfate, (**c**) tyrindoleninone,(**d**) 6-bromoisatin, and (**e**) 6,6′dibromoindirubin.

The study aims to further evaluate the anti-inflammatory drug potential of some secondary metabolites derived from *D. orbita*—tyrindoxyl sulfate, tyrindoleninone, 6-bromoisatin, and 6,6′dibromoindirubin (Figure 1)—through virtual screening (molecular docking) and to decipher their ligand–protein interaction with COX-1/2. Molecular dynamics simulation experiments and binding energy calculations were performed to identify the stability and compactness of the selected ligand–protein complex. Comparative analysis was performed against aspirin (Figure 1a), the selected FDA-approved, widely used, and oldest anti-inflammatory lead molecule [38,39]. In addition, we characterized their pharmacokinetic and toxicokinetic profiles to predict the bioactivity and safety of these brominated indoles.

## 2. Results and Discussion

### 2.1. Molecular Docking Analysis

**Figure 2:**
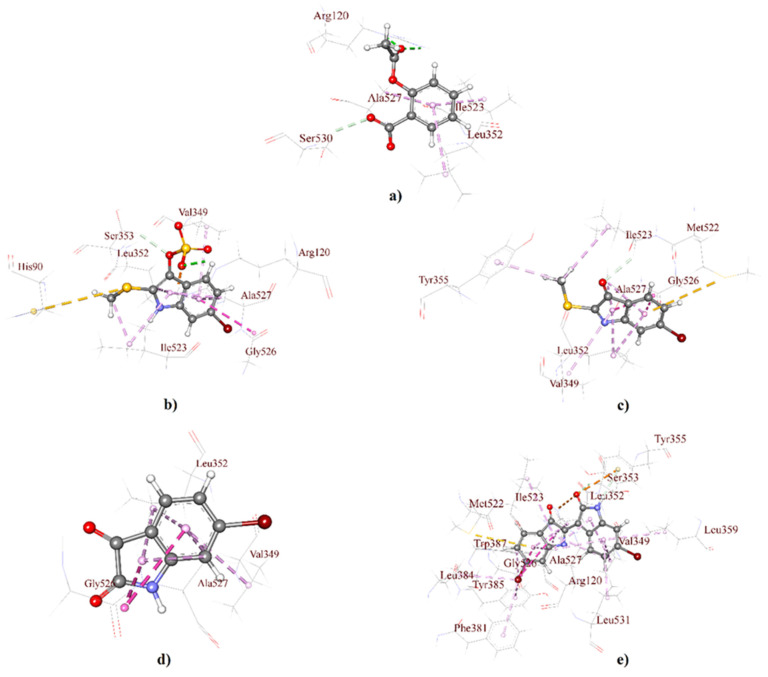
3D interaction maps (distances Å) of *Dicathais orbita* brominated indole derivatives and standard aspirin showing the crystallographic ligand with a COX-1 active binding site; (**a**) aspirin, (**b**) tyrindoxyl sulfate, (**c**) tyrindoleninone, (**d**) 6-bromoisatin, and (**e**) 6,6′-dibromoindirubin.

**Figure 3:**
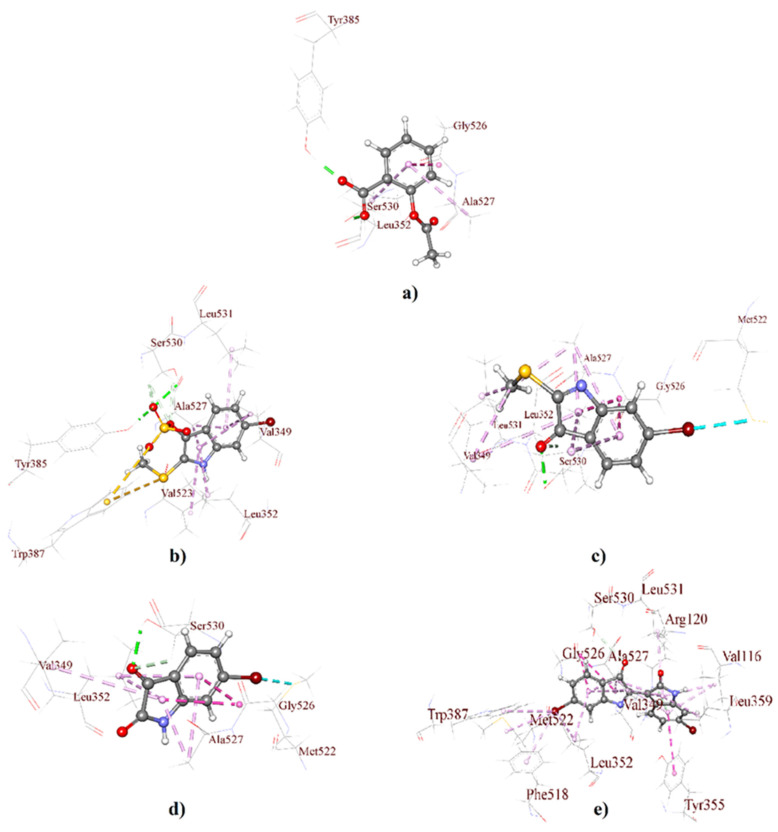
3D interaction maps (distances Å) of *Dicathais orbita* brominated indole derivatives and standard aspirin showing the crystallographic ligand with a COX-2 active binding site; (**a**) aspirin, (**b**) tyrindoxyl sulfate, (**c**) tyrindoleninone, (**d**) 6-bromoisatin, and (**e**) 6,6′-dibromoindirubin.

Molecular docking is a standard approach for structure-based drug design to evaluate the atomic level interaction between small molecules and a protein; thus, it helps to identify target specificity along with binding affinity [40,41,42]. Molecular docking studies, employed here through GLIDE, predict the binding affinity of the 3D structure of *D. orbita* secondary metabolites into a cyclooxygenase isoform COX-1 (Figure 2) and COX-2 binding site (Figure 3). The outcomes of the GLIDE scores, GLIDE energy, GLIDE model, and GLIDE ligand from the docking analyses are presented in Table 1 and Table 2 for COX-1 and COX-2, respectively. As shown in Table 1, the docking score range for the mollusk brominated indoles was −6.06 to −7.25 kcal/mol for COX-1, which is comparatively better than the reference compound aspirin (−2.80 kcal/mol). On the other hand, the docking score of aspirin was −6.87 kcal/mol with the COX-2 enzyme, which was similar for the indole derivatives tyrindoxyl sulfate (−6.34 kcal/mol) and 6-bromoisatin (−6.19 kcal/mol). Moreover, tyrindoleninone showed a high binding affinity toward COX-2, with a docking score of −7.17 kcal/mol. Interestingly, 6,6′ dibromoindirubin exhibited a high binding affinity to COX-1, and the docking score was −7.25 kcal/mol, whereas the docking score of this compound was only −3.14 kcal/mol for COX-2.

The 3D receptor–ligand interactions are illustrated for each compound as a protein–ligand interaction diagram for COX-1 (Figure 2) and COX-2 (Figure 3). The specific details of the non-bond interactions for all *D. orbita* compounds, their bond category, types, amino acids, ring or atoms, and distance involved in the inhibition are detailed in Table 1 (Table S1) and Table 2 (Table S2) for COX-1 and COX-2, respectively.

Selectivity towards COX-2 is usually preferred for anti-inflammatory agents to minimize the potential side effects [43]. The structural differences between the binding sites of COX-1 and COX-2 offer valuable strategies for the design of selective COX-1/2 inhibitors [44,45,46]. The cyclooxygenase active site for prostaglandin synthesis is found deep inside a pocket with 19 amino acid residues within cell membranes, permitting easy access for insoluble arachidonic acid [47,48]. All the secondary metabolites studied here significantly bind within the key pocket, showing a close distance (Å) and interaction with the active amino acid residue Serine-530 (Ser-530) via hydrogen bonds (Figure 3, Table S2). Notably, aspirin, the first NSAID, covalently alters both COX-1 and COX-2 through the acetylation of amino acid residue Ser-530 and inhibits cyclooxygenase activity [49,50,51] by preventing the appropriate binding of arachidonic acid [50,52]. 

Aspirin and other aspirin-like substances, known to inhibit prostaglandin synthesis and release, including indomethacin and indomethacin analog sulindac, interact with COX via multiple amino acids. For example, the indole ring of indomethacin and sulindac showed the interaction with amino acid residue Valine-349 (Val-349) [53,54]. The hydroxyl of Ser-530, along with Val-349, in COX-1 and -2 appears to be crucial for the production of prostaglandin G2 (PGG2) [55,56,57]. It is, therefore, noteworthy that the *D. orbita* secondary metabolites, also derived from the heterocyclic compound indole, show pi-alkyl hydrophobic interactions with the active amino acid residue Val-349 for both COX-1/2 (Figure 2 and Figure 3, Tables S1 and S2)**,** providing further support for the likely inhibition of COX by these marine compounds.

The brominated indole derivatives tested from *D. orbita* exhibited amide pi-stacked, alkyl, pi-alkyl, types of hydrogen, hydrophobic, electrostatic, and halogen interactions with the amino acid residues in COX-1 and 2, similar to that observed in standard NSAID acetylsalicylic acid or aspirin (Tables S1 and S2). In particular, the present docking study showed that tyrindoxyl sulfate, the ultimate precursor of the Tyrian purple pigment, interacts with glycine-526 (Gly-526), alanine-527 (Ala-527), leucine-352 (Leu-352), arginine-120 (Arg-120), tyrosine-385 (Tyr-385), serine-353 (Ser-353), tryptophan-387 (Trp-387), leucine-531 (Leu-531), and isoleucine-523 (Ile-523) (Figure 2b and Figure 3b), whereas the methylthio group of tyrindoleninone also interacts with Gly-526, Ala-527, Leu-352, and Tyr-355, along with Leu-531, Ile-523, and methionine-522 (Met-522) (Figure 2c and Figure 3c). 6-Bromoisatin, which is a precursor of the red Tyrian purple isomer 6,6′dibromoindirubin, also exhibited interaction with Gly-526, Ala-527, Leu-352, and Met-522 (Figure 2d and Figure 3d). Additionally, 6,6′dibromoindirubin interacts with Gly-526, Ala-527, Leu-352, Arg-120, Tyr-385, Ser-353, Trp-387, Leu-531, Ile-523, Tyr-355, phenylalanine-381 (Phe-381), phenylalanine-518 (Phe-518), and Met-522 (Figure 2e and Figure 3e). Notably, Gly-526, along with Leu-384 in COX, controls the carbon ring cyclization in prostaglandin biosynthesis [58], whereas the neighboring Leu-352 increases the pocket size for cyclooxygenase activity [44,45,59]. Consequently, Leu-352, in the active site pocket of COX, is a known anti-inflammatory target that has been previously reported to interact with heterocyclic compounds [20,60,61]. Furthermore, Arg-120, along with the catalytically significant residue Tyr-385, is known as the aliphatic backbone of the cyclooxygenase active site [62,63,64]. Arg-120, which is placed about midway along the apex and entrance of the active site, binds to the carboxylate groups of many NSAIDs and fatty acids, whereas Tyr 385, in its radical form, reduces arachidonic acid during its conversion to prostaglandin G2 (PGG2) [65,66,67]. Consequently, the interaction of the mollusk compounds with Arg-120, Tyr-385, and Leu-352 in the active binding site of COX is likely to interfere with prostaglandin biosynthesis.

On the other side, the amino acid residues Leu-531 and Ile-523 exhibit conformational flexibility at the entrance of the cycloxygenase channel [43,68,69]. However, the pragmatic elasticity for the Leu-531 side chain is exclusive to COX-2 [64]. Nevertheless, 6,6′dibromoindirubin, which showed a lower binding affinity to COX-2, was found to interact with these amino acids. However, unlike the other *D. orbita* compounds, 6,6′dibromoindirubin was found to interact with Phe-318 and Phe-518. Phe-318 is thought to show measurable contributions towards optimizing cyclooxygenase catalysis [56], whereas Phe-518 increases the volume of the COX-2 NSAID binding location by ~20% over that in COX-1, which affords access to COX-2 selective inhibitors [19,70]. Met-522, along with Phe-518, contributes to the foremost shell of the cyclooxygenase hydrophobic channel [56]. NSAIDs, like meloxicam, can form hydrogen bonding interactions through Met-522 and Trp-387 at the apex of the active site of cyclooxygenase [20]. Several of the *D. orbita* compounds, including 6,6′dibromoindirubin, were found to interact with these two amino acids.

Overall, the *D. orbita* brominated indoles interact with multiple amino acids in the COX-1 and 2 binding sites, with further validation performed through the molecular dynamics simulations.

### 2.2. Molecular Dynamics Simulation Analysis

#### 2.2.1. Root Mean Square Deviation (RMSD)

**Figure 4:**
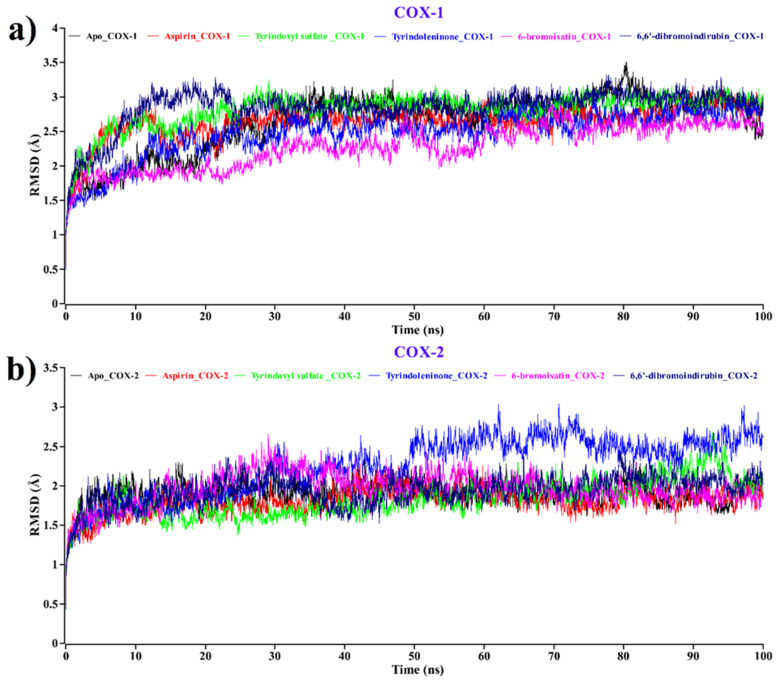
Time evolution of root mean square deviation (RMSD) for the protein of each docked complex for (**a**) COX-1 and (**b**) COX-2. Complexes: Black—apo protein, red—aspirin, green—tyrindoxyl sulfate, blue—tyrindoleninone, magenta—6-bromoisatin, navy blue—6,6′-dibromoindirubin.

The atomic RMSDs of the Cα atoms for a protein–ligand complex of aspirin (red) and tyrindoxyl sulfate (green), tyrindoleninone (blue), 6-bromoisatin (magenta), and 6, 6′-dibromoindirubin (navy blue) were calculated and plotted in a time-dependent manner along with the Apo form (black) of the COX- 1/COX-2 protein (Figure 4).

In Figure 4a, the plot demonstrates that when complexed with COX-1, all the *D.orbita* compounds, along with aspirin, show a stable nature, such as the Apo form of COX-1. On the other hand, in Figure 4b, tyrindoleninone (blue) remained stable from 0 to 49 ns, showing an average 2 Å RMSD value and, after that, revealing some small fluctuations in its backbone structure. After 50 ns, it showed a stable form. In Figure 4b, it is indicated that all compounds and aspirin bound to COX-2 show a similar stable pattern to the Apo form of COX-2. From this analysis, it can be inferred that upon the binding of tyrindoxyl sulfate (green), tyrindoleninone (blue), 6-bromoisatin (magenta), and 6,6′-dibromoindirubin (navy blue) compounds to COX-1 and COX-2, there was no change in the stability of both proteins (Figure 4).

#### 2.2.2. Radius of Gyration (Rg)

**Figure 5:**
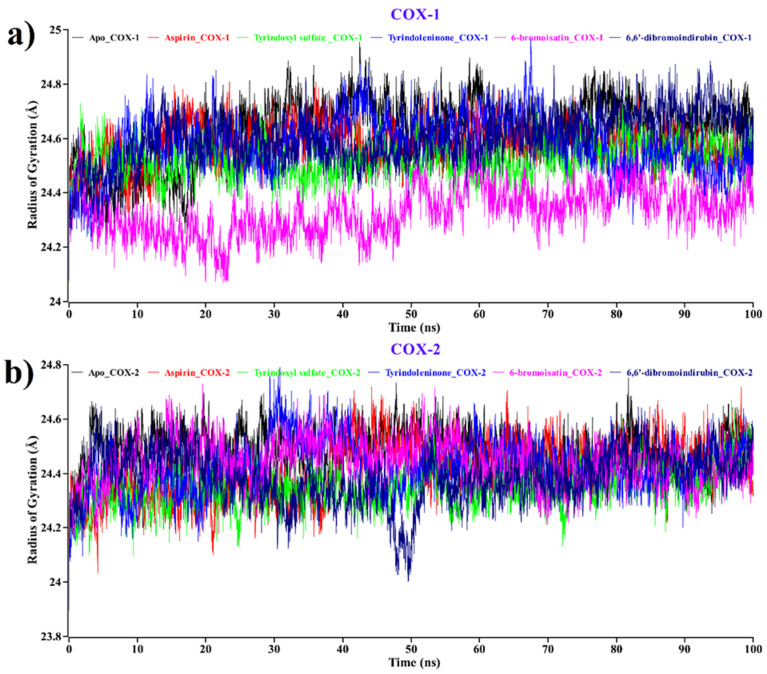
Time evolution of the radius of gyration for the protein of each docked complex for (**a**) COX-1 and (**b**) COX-2. Complexes: Black—apo protein, red—aspirin, green—tyrindoxyl sulfate, blue—tyrindoleninone, magenta—6-bromoisatin, navy blue—6,6′-dibromoindirubin.

We also concluded the Rg value analysis for both apo proteins, aspirin, and compounds (Figure 5) to study the influence of ligand binding to protein in terms of compactness [71,72]. Lesser Rg values suggest good compactness between ligand and protein, where the stably folded protein shows a consistent Rg value. The Rg value changes by degrees with the change of structure of the protein.

The average Rg value for the apo form of the COX-1 protein (black) was 24.62 Å (Figure 5a). On the other hand, aspirin (red), tyrindoxyl sulfate (green), tyrindoleninone (blue), 6-bromoisatin (magenta), and 6, 6′-dibromoindirubin (navy blue) were shown to have 24.57, 24.52, 24.57, 24.32, and 24.60 Å on average Rg value. Here, tyrindoleninone shows the same pattern of Rg value as aspirin, while 6-bromoisatin shows a decreased Rg value throughout the duration of the experiment. According to Figure 5b for COX-2, it can be predicted that the average Rg value for the apo form of protein (black) was 24.48 Å. On the other hand, aspirin (red), tyrindoxyl sulfate (green), tyrindoleninone (blue), 6-bromoisatin (magenta), and 6, 6′-dibromoindirubin (navy blue) showed 24.41, 24.35, 24.41, 24.44, and 24.38 Å on average Rg values. Surprisingly, for COX-2, tyrindoleninone again shows the same pattern of Rg value as aspirin, while tyrindoxyl sulfate showed the most decreasing Rg value among all.

Hence, by revealing a lower Rg value, this analysis indicates better compactness and a healthy binding pattern for all our compounds against COX-1 and COX-2.

#### 2.2.3. Solvent Accessible Surface Area (SASA)

**Figure 6:**
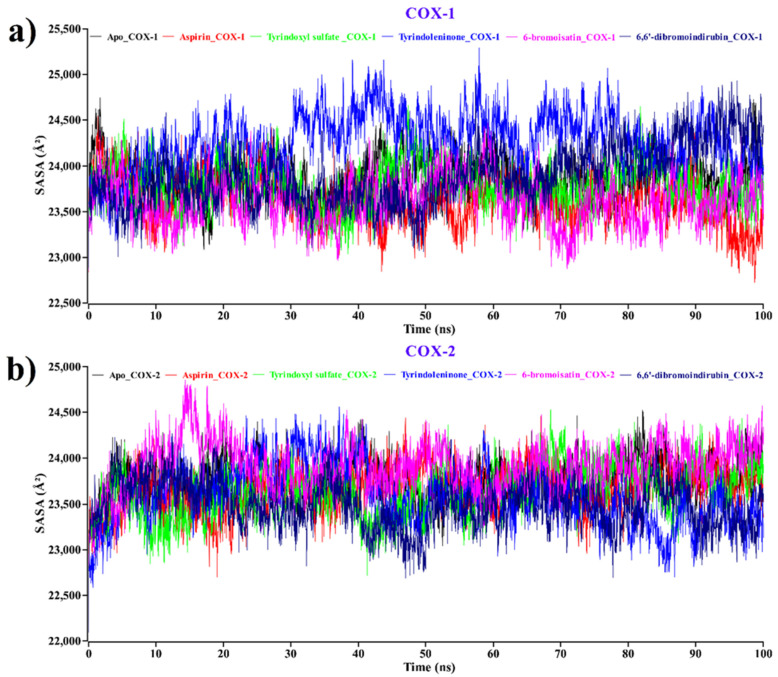
Time evolution of SASA for the protein of each docked complex for (**a**) COX-1 and (**b**) COX-2. Complexes: black—apo protein, red—aspirin, green—tyrindoxyl sulfate, blue—tyrindoleninone, magenta—6-bromoisatin, navy blue—6,6′-dibromoindirubin.

The SASA of a protein is explored as a crucial factor in protein stability and compactness in protein folding studies [73]. The SASA values for the apo form of COX-1 and COX-2, as well as the proteins complexed with each of the compounds, along with aspirin, were calculated, and the outcomes are illustrated in Figure 6.

The average SASA values for apo-COX-1 (black), aspirin (red), tyrindoxyl sulfate (green), tyrindoleninone (blue), 6-bromoisatin (magenta), and 6, 6′-dibromoindirubin (navy blue) were 23,842, 23,634, 23,788, 242,67, 23,617, and 23,886 Å², respectively (Figure 6a). On the other hand, the average SASA values for apo-COX-2 (black), aspirin (red), tyrindoxyl sulfate (green), tyrindoleninone (blue), 6-bromoisatin (magenta), and 6, 6′-dibromoindirubin (navy blue) were 23,773, 23,669, 23,629, 23,586, 23,904, and 23,479 Å², respectively (Figure 6b). The average SASA value showed that all four compounds had a similar pattern of SASA values compared to the Apo form of COX-1 and COX-2 proteins. From the SASA values, we have concluded that the binding of all compounds induced conformational stability and better compactness during the binding with apo-COX-1/COX-2.

#### 2.2.4. Root Mean Square Fluctuations (RMSFs)

**Figure 7:**
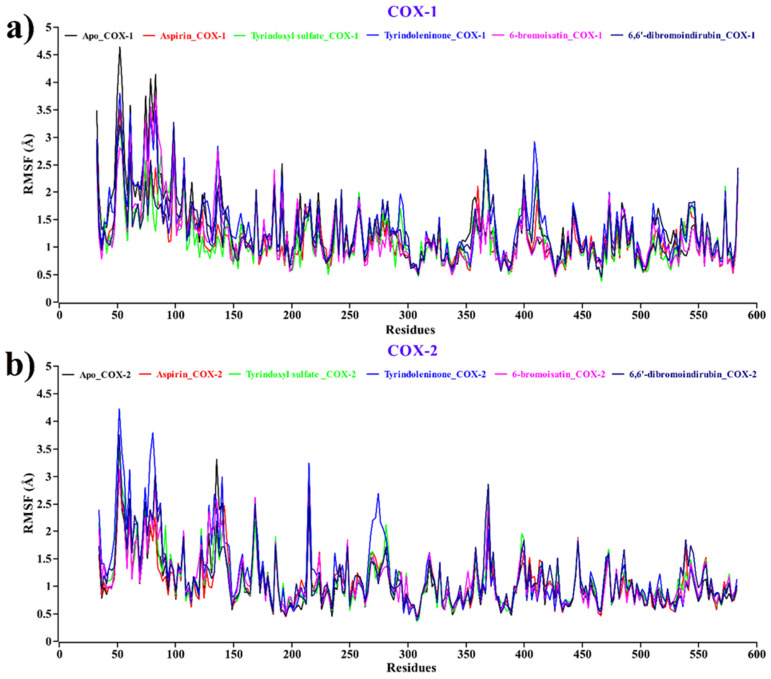
Root mean square fluctuations of protein–ligand of each docked complex for (**a**) COX-1 and (**b**) COX-2. Complexes: Black—apo protein, red—aspirin, green—tyrindoxyl sulfate, blue—tyrindoleninone, magenta—6-bromoisatin, navy blue—6,6′-dibromoindirubin.

Root mean square fluctuation (RMSF) values of different compounds and aspirin, along with the Apo form of COX-1/2, have been calculated at every trajectory of molecular dynamics simulation to evaluate the dynamic behavior of the complexes since it estimates the flexibility of local amino acids in the complex. In this RMSF plot (Figure 7), peaks demonstrate the areas of the protein that fluctuated most in the entire simulation period.

The average RMSF values for the apo protein (black) as well as aspirin (red), tyrindoxyl sulfate (green), tyrindoleninone (blue), 6-bromoisatin (magenta), and 6,6′dibromoindirubin (navy blue) for both COX-1 and COX-2 were 1.325, 1.153, 1.147, 1.364, 1.192, and 1.249 Å and 1.056, 1.085, 1.123, 1.225, 1.127, and 1.145 Å, respectively. From the RMSF plot, it can be seen that of all the compounds, aspirin, along with Apo, have the lowest and highest root means square fluctuations at the same amino acid residue with the same position. The highest fluctuations have been observed (Figure 7a,b) in several regions, ranging from 50–60, 80–100, 350–380, and 400–420 for both COX-1 and COX-2, respectively.

### 2.3. MM–PBSA Binding Free Energy Analysis

**Figure 8:**
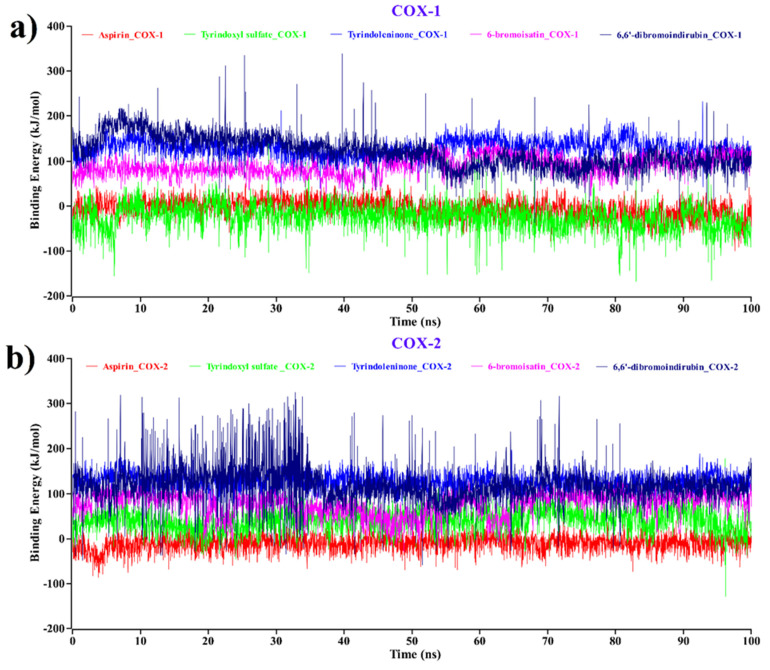
Binding free energy (in kJ mol^−1^) of each snapshot was calculated by molecular mechanics–Poisson–Boltzmann surface area (MM–PBSA) analysis, representing the change in binding stability of each docked complex for (**a**) COX-1 and (**b**) COX-2. Complexes: red—aspirin, green—tyrindoxyl sulfate, blue—tyrindoleninone, magenta—6-bromoisatin, navy blue—6,6′-dibromoindirubin.

The molecular mechanics–Poisson–Boltzmann surface area (MM–PBSA) method has been generally used as a reliable and efficient free energy simulation approach to calculate the binding energy of protein–ligand complexes [74,75,76]. To understand the binding ability of the ligands towards its receptor, the interpretation of binding free energy is necessary [77,78]. In view of this, we exposed each protein–ligand complex of COX-1 and COX-2 to the MM–PBSA binding energy calculation to investigate structural changes during ligand binding; the results are plotted in Figure 8, where the more positive energy values indicate better binding [79,80].

According to Figure 8a, for COX-1 complexes, the average values of the binding free energy of tyrindoxyl sulfate (green), tyrindoleninone (blue), 6-bromoisatin (magenta), and 6,6′dibromoindirubin (navy blue) were -24.216, 128.936, 89.899, and 120.13 kJ/mol, respectively. The aspirin–COX-1 complex shows a -5.818 kJ/mol binding free energy value. This demonstrates that all the compounds except tyrindoxyl sulfate bind effectively to COX-1 and show higher binding energy compared to aspirin–COX-1.

On the other hand, in Figure 8b, for aspirin–COX-2, the binding energy shows negative values (average = −10.46 kJ/mol). Comparing the averages, the binding free energy values of tyrindoxyl sulfate, tyrindoleninone, 6-bromoisatin, and 6,6′dibromoindirubin with COX-2 complexes were all positive, with averages of 41.278, 126.978, 77.051, and 117.768 kJ/mol, respectively. Tyrindoxyl sulfate, which showed negative binding energy when complexed with COX-1 (Figure 8a), interestingly showed positive binding energy values with COX-2 (Figure 8b)**,** indicating the potential for the selective inhibition of COX-2. 

A large difference in the binding energy of tyrindoleninone, 6-bromoisatin, and 6,6′dibromoindirubin complexes was also observed compared to aspirin for COX-1/2, indicating tighter binding. Notably, a steady nature has been observed for the complexes with tyrindoleninone and 6-bromoisatin, without any significant fluctuations.

From the 100 ns molecular dynamics (MD) simulation, we can conclude that RMSD, Rg, SASA and RMSF analyses validate the binding of *D. orbita* compounds, observed from molecular docking against COX-1/2.

The RMSD analysis demonstrated that upon the binding of these brominated indoles to the COX-1/COX-2, there was no change in the stability of the proteins. RMSF, Rg, and SASA analyses also revealed a strong binding pattern for tyrindoxyl sulfate, tyrindoleninone, 6-bromoisatin, and 6,6′dibromoindirubin with COX-1/COX-2.

Moreover, binding free energy analysis also revealed excellent results with tyrindoleninone, 6-bromoisatin, and 6,6′dibromoindirubin complexes towards COX-1/2 and tyrindoxyl sulfate for COX-2, showing higher binding energy values compared to the aspirin complex and representing better binding affinity and stable complex formation, consistent with the conclusion of the RMSF, Rg, and SASA analyses.

### 2.4. Physicochemical Properties and Drug-Likeness

**Figure 9:**
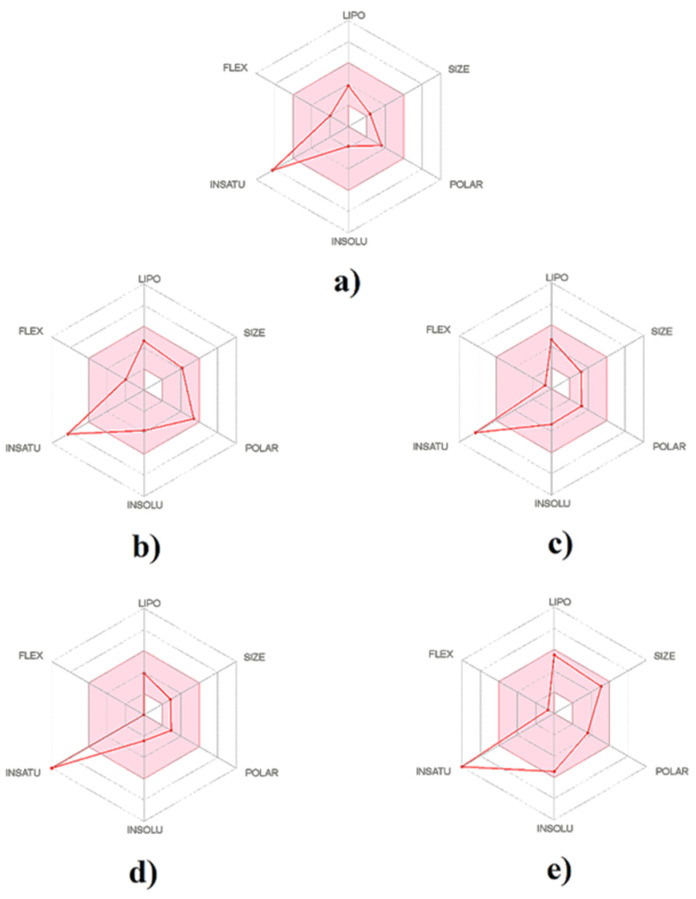
The bioavailability radar of brominated indole derivatives from *Dicathais orbita* compared to the NSAID control aspirin, generated using the SwissADME web tool: (**a**) aspirin, (**b**) tyrindoxyl sulfate, (**c**) tyrindoleninone, (**d**) 6-bromoisatin, and (**e**) 6,6′dibromoindirubin.

The physicochemical properties, as well as drug-likeness of *D. orbita* secondary metabolites, were determined through SwissADME web-based tools, as described previously by Diana et al. [81], and the output values are summarized in Table 3. The bioavailability radar offers a graphical picture of the drug-likeness parameters (Figure 9). Principle coordinate ordination highlights the differences in physicochemical parameters between the brominated indole ligands and aspirin, with molecular weight and heavy aromatic atoms driving separation along PC1 and the polar surface area, influencing the separation on tyrindoxyl sulfate along PC2 (Figure S1).

Drug-likeness and physicochemical properties are a composite of molecular properties and structural features that regulate whether a molecule has features compatible with drug absorption by comparison with recognized drugs that are known to alter biological function [82,83]. Twelve molecular properties, along with Lipinski’s rule, which is vital for evaluating the drug-likeness for oral bioavailability of a molecule, were considered for the *D. orbita* secondary metabolites (Table 3). According to Lipinski’s rule, most “drug-like” molecules have an octanol–water partition coefficient [84] (log P) that does not exceed 5, molecular weight <500, number of hydrogen bond acceptors <10, and number of hydrogen bond donors <5. Notably, all the secondary metabolites of *D. orbita* passed the filter of Lipinski’s rule with zero violation, which is consistent with previous findings reported by [33].

As shown in Table 3, all the *D. orbita* ligands are within the range for molar refractivity (MR) and topological polar surface area (TPSA), according to the range set by the SwissADME web tool [81]. This result is also consistent with the formula of Lorentz–Lorenz, which relates molecular weight, molar refractivity, and polar surface area [85]. Notably, MR denotes the molar volume modified by the refractive index, which characterizes the size and polarizability of a molecule or fragment [86]. The polar surface area (PSA) is designed using the fragmental technique termed TPSA, considering sulfur as a polar atom [87], which therefore contributes to the polar surface area of tyrindoxyl sulfate and tyrindoleninone.

The oral availability of the studied indole compounds is illustrated in the bioavailability radar plots (Figure 9). This demonstrated that the bioavailability radar for all the brominated indoles tested was similar to aspirin (Figure 9) and within a suitable range of oral bioavailability. It was found that all the brominated compounds, along with aspirin, are slightly outward of the pink region on one edge, which represented the fraction of carbon bond saturation (Csp3). The carbon bond saturation is identified as the number of sp3 hybridized carbons/total carbon count, and the descriptor is associated with solubility and melting point [88]. 

Lipophilicity is a crucial physicochemical property for pharmacokinetic drug discovery [89,90]. From the log *p*-values (Table 3), it can be concluded that the brominated indoles are predicted to have good lipophilic characters and are within a suitable range of water solubility using log S (ESOL) values [91], representing the compounds that are moderately water-soluble (Table 3). A range of lipophilicity calculations is available based on the ratio of octanol solubility to aqueous solubility [92]. iLOGP considers the free energy of solvation according to the solvent-accessible surface area (GB/SA) model established by Daina et al. [93], whereas XLOGP3 is an atomistic system, including a knowledge-based library and corrective factors [94]. M-LOGP uses an archetype of topology, relying on a linear affiliation with 13 molecular descriptors [95,96], and SILICOS-IT is a hybrid technique, depending on seven topological descriptors and 27 fragments [81]. Using all of these predictors of lipophilicity, the *D. orbita* brominated indoles were predicted to be in a suitable range of drug absorption and to share comparable values with standard aspirin. Notably, there is a general consensus that the drug-like properties are linked with pharmacokinetic and toxicological properties [97,98].

### 2.5. Pharmacokinetics and Toxicological Properties

The clinical progress of drugs to the market is only approximately 20% [99,100,101] due to the low percentage of compounds with suitable pharmacokinetic and toxicokinetic properties. Problems include poor absorption, high elimination rate, and hepatic clearance due to low bioavailability [102,103,104]. Therefore, absorption, distribution, metabolism, excretion, and toxicity (ADMET) descriptors of a chemical entity should be investigated early in drug development to comprehend the required safety and potential potency evidence for regulatory approval [105,106]. ADMET profiles for all compounds were evaluated using Qikprop (Schrödinger, LLC, New York, NY, USA) and pkCSM (University of Melbourne, Vic, Australia) databases. Table 4 illustrates the relative ADMET profiles of the four *D. orbita* compounds compared to aspirin as a standard.

All the brominated indoles tested here are predicted to have better absorption into the intestine than aspirin (Table 4). This result is consistent with in vivo studies in a rodent model for colorectal cancer using extracts of these brominated indoles from *D. orbita*, where desorption/ionization on porous silicon–mass spectrometry imaging (DIOS–MSI) revealed the availability of the brominated metabolites in the GI tract [107].

Compounds are considered to have a high human epithelial colorectal adenocarcinoma (Caco-2 cells) permeability if they have a Papp > 10 × 10^−6^ cm/s (80−100% Fa) [108], equivalent to >0.90 in the pkCSM server [109]. All the secondary metabolites of *D. orbita* have high Caco-2 cell permeability except 6,6′ dibromoindirubin (Table 4). Nevertheless, the Caco-2 cell permeability value of 6,6′ dibromoindirubin is still substantially higher than aspirin (Table 4). It is important to note that 6-bromoisatin and tyrindoleninone, which are predicted to have high Caco-2 permeability, are target compounds for the prevention of colorectal cancer. These brominated indoles effectively reduced cell viability and induced apoptosis in two human colon adenocarcinoma cell lines, HT29 and Caco2 [110], as well as induced apoptosis in DNA-damaged cells of the colon in vivo [109,111]. Caco-2 cells are most frequently used in intestinal permeability models, and they have been validated for drug absorption studies [112]. Notably, the COX-2 isoenzyme has been demonstrated to play a vital role in the progression of colorectal cancer through the elevation of angiogenesis, anti-apoptotic effects, and increased invasiveness [113]. Several in vitro, in vivo, and clinical studies have substantiated that COX-2 inhibitors help to prevent colorectal cancer [114,115]. This further supports the potential for these molluscan brominated indoles to be developed as colorectal cancer treatments due to their predicted COX inhibition properties, along with Caco-2 cell permeability.

Moreover, it has been found that none of the *D. orbita* secondary metabolites acted as P-glycoprotein (P-gp) inhibitors (Table 4). P-gp is a plasma membrane protein that performs as a confined drug transport mechanism, dynamically extruding toxins and xenobiotics out of the cells, and it plays an extensive role in drug absorption and disposition [116,117]. The effects of P-gp on the distribution, metabolism, and excretion of drugs, along with a potential transport role in different organs, such as the liver, kidney, pancreas, uterus, placenta, small intestine, and colon, in the body is well established [118,119]. The lack of activity against this key transporter protein supports the safety of the brominated indoles from *D. orbita*.

After being absorbed into the circulatory system, drugs move reversibly between different compartments within the body, dictating their biodistribution [120]. The plasma protein binding (QPlogKhsa) values for distribution showed that all the four *D. orbita* compounds are within the recommended range (−1.5 to 1.5, Table 4) [121]. Plasma protein-binding influences the absorption, distribution, metabolism, and excretion (ADME) of small molecules [122,123]. In addition, the blood–brain barrier (BBB) value corresponds to the ability of a compound to enter the central nervous system. The range of BBB values for a drug candidate should be between −3.0 to 1.2 [109,124]. All the studied brominated compounds have a BBB value below this range except 6-bromoisatin (Table 4). It is noteworthy that isatin is known as an endogenous indole, with diverse distribution in the brain as well as tissues. The concentration of isatin in the brain, as well as in the hippocampus and cerebellum, is predominantly high, at levels of about 0·1 μg/g, where it acts as a modulator of biochemical action [125,126]. Additionally, microglial cells, the macrophages of brain parenchyma, are the key players of the brain’s innate immune response. Microglia are an important source of prostaglandins (PGs), and they are responsible for certain neuroinflammatory diseases [127], which are also important targets of NSAIDs within the brain [128]. NSAIDs can act constructively in diseases such as epilepsy, Alzheimer’s disease, or traumatic brain injury, for which modifications of BBB functionality are necessary [129,130,131,132,133]. Hence, the isatin derivative 6-bromoisatin, which is projected to inhibit the COX enzyme as well as cross the BBB, should be further investigated for the treatment of neuroinflammatory diseases.

Drug metabolism enzymes are critical factors for drug bioavailability. The cytochrome P450 enzyme (CYP450) is one of the crucial hepatic enzymes, responsible for most of the drug metabolism [134]. CYP2D6 is one of the major subtypes of cytochrome P450 [135]. The potential metabolism of *D. orbita* brominated indoles through the CYP2D6 enzyme was investigated on the pkCSM server [109]. None of the ligands were found to be a substrate or inhibitor of this hepatic enzyme (Table 4).

The drug elimination process, also known as drug clearance, generally includes liver metabolism and excretion, where the kidneys play vital roles for drug elimination [136]. The rate of clearance of the *D. orbita* secondary metabolite was projected to be low in comparison with aspirin (Table 4). It is noteworthy that organic cation transporter 2 (OCT2) plays an important role in the uptake and disposition of the renal clearance of drugs [137,138]. The compounds here are not likely to be OCT2 substrates, except 6,6′ dibromoindirubin (Table 4). Notably, in previous in vivo research, a diuretic effect has been observed for 6-bromoisatin [139], and 6,6′dibromoindirubin was observed to form in the gastrointestinal tract of mice treated with 6-bromoistain via oral administration [107].

Attrition due to clinical side effects and toxicity is a major concern in drug discovery [140,141]. Interestingly, all the *D. orbita* compounds were exempted from hepatotoxicity, and not a single one of these compounds was found to be mutagenic as per AMES toxicity (Table 4). The oral acute toxicity LD_50_ in rats is predicted to be comparable to or below aspirin. None of the *D. orbita* compounds were projected for *human ether-a-go–go gene (hERG) I* inhibition. Notably, oral administration of *D. orbita* extract containing these brominated indoles did not show evidence of major clinical toxicity during in vivo toxicity evaluation [142], although some weak idiosyncratic effects were observed in the liver as well as in the gastrointestinal tract, which could be due to other compounds or artifacts in extracts. Early identification of toxicity is important for the evaluation of the potentiality of a drug candidate [143], and the results presented here are promising for the further development of brominated indole derivatives.

### 2.6. Modelling Biological Predictions to Physicochemical Properties

To provide some insight into the physicochemical properties that influence the strength of COX-1 and 2 binding interactions with the brominated indoles, distance-based linear modeling was undertaken. This revealed some differences in the individual properties influencing the binding of the brominated indoles to COX-1 and 2 (Table 5). COX-1 binding was influenced by aqueous solubility and total polar surface area as well as molecular weight and molar refractivity, whereas COX-2 binding was decreased by the aromatic heavy atoms and high logP of 6,6 dibromoindirubin (Table 5a). Previous quantitative structure–activity relationship studies on heterocyclic compounds have highlighted the importance of hydrophilic interactions at the binding site of COX-2 as well as the size, shape, and molecular refractivity for selective COX-1/2 inhibition [144].

Regression models for the predicted pharmacokinetic and toxicokinetic factors of the brominated indoles revealed no significant relationship with individual physicochemical variables for intestinal absorption, tissue permeability, clearance, or oral toxicity (Table 5a). However, the combinations of physicochemical parameters explained a high proportion of the variation in the predicated biological properties for these brominated indoles, with lipophilicity (logP) featuring as a contributing factor in all cases (Table 5b). Higher absorption and permeability in the intestine are expected with higher logP values, and simultaneously higher logP values are expected to lower renal clearance due to lower plasma protein binding [145]. Well-balanced pharmacokinetics based on physicochemical properties has been previously reported for anti-inflammatory indole derivatives [146] and is likely to contribute to their bioavailability as oral drugs. Molecular weight was also identified as an important factor influencing the variation in intestinal absorption and CaCo-2 permeability, whereas the ratio of sp3 hybridized carbons impacted permeability across the blood–brain barrier, total clearance, and oral toxicity. These data provide further insights into the structural features of brominated indoles that could influence their biodistribution and in vivo bioactivity, future drug design, and optimization. 

## 3. Materials and Methods

### 3.1. Preparation of Ligand

The 3D structure of the ligands (*D. orbita* compounds) and standard aspirin were obtained from the PubChem website (https://pubchem.ncbi.nlm.nih.gov accessed 1 November 2019) in sdf format and then imported into the Maestro (Schrödinger, LLC, New York, NY, USA) molecular modeling platform. The structures were introduced into the job table, and the ligands were organized using the software Ligprep from the Schrödinger suite 2018, Maestro v11.6 (Masetro, Schrödinger, LLC, New York, NY, USA) [147]. Primarily, the ligands were presented in simplified molecular-input line-entry system (SMILES) strings. A single small energy 3D conformer for the individual structure was produced, with tautomers and ionization states in the pH range 7.4 ± 0.2, continued by optimized potentials for liquid simulation (OPLS3e) force field optimization. The Macro Model module was introduced in the Schrödinger package, using the default settings for charge calculation [147]. 

### 3.2. Preparation of Protein

The X-ray crystallographic 3D structures of COX-1 (PDB code: 3N8X, resolution 2.75Å), crystallized by Sidhu et al. [148], and COX-2 (PDB code: 5IKR, 2.34 Å), formed by Orlando and Malkowski [149], were downloaded from the online Protein Data Bank (RCSB PDB https://www.rcsb.org/ accessed 1 December 2019–20 January 2020). The active site was selected for docking experiments and processed in Maestro through the protein preparation wizard facility [148]. The subsequent preparation stages were concluded: (i) protein structure integrity was verified, and missing residues were included using (ii) prime bond orders assigned and hydrogen atoms attached to the ligand molecule; (iii) protein heavy atoms merged with hydrogen atoms; (iv) side chain optimization, along with hydroxyl group orientation and (v) the state of residues determined. Throughout the protein preparation process, the ligand was retained. Lastly, the COX-1/2–ligand complexes were appointed to geometry refinement using OPLS3e force field restrained minimization.

### 3.3. Grid Generation

Receptor grid generation was performed by Glide (grid-based ligand docking with energetics) of Schrodinger–Maestro version 11.6 (LLC, New York, NY, USA). Here, van der Waals radius scaling was set to the default scaling factor of 1.00 Å and charge cut-off of 0.25 Å. A cubic box of particular dimensions was set on the centroid of the active site residues, where they were created for the receptor. The bounding box was fixed to 10 × 10 × 10 Å to identify the dynamic binding site in the target protein.

### 3.4. Molecular Docking Studies

Molecular docking was employed to evaluate the affinity of the binding of *D. orbita* secondary metabolites towards cyclooxygenase isoform COX-1 and COX-2 binding sites. Glide flexible ligand docking was used here for docking studies [150,151], within which penalties were implemented to non-*cis/trans* amide bonds. Glide XP extra precision docking was also applied, keeping all docking factors as default. No bonding restraints were provided during docking calculations. Using the Monte Carlo algorithm, ligand poses (by “pose”, we mean a full description of the ligand: orientation and position relative to the receptor as well as core conformations) were produced for individual input molecules, and the ligand efficiency of these molecules to the COX-1/2 enzymes was predicted using the Glide docking score. 

### 3.5. Molecular Dynamics Simulation

The predictions from molecular docking studies were validated using molecular dynamics simulation using YASARA Dynamics software [152]; the settings for molecular dynamics simulation were adapted from Uzzaman et al. [153], with some modifications. The AMBER14 force field [154] was used for this study, which is extensively used to explain the macromolecular system. Additionally, the transferable intermolecular potential 3-point (TIP3P) water model was employed by adding Cl^−^ and/or Na^+^ ions, where the entire solvent molecules were 92,657 with a density of 0.997 gm/cm^3^. To carry out the simulation, the periodic boundary requirement was incorporated, with the box size 90 × 90 × 90 Å3. The minimization of initial energy for each simulation system was conducted by the simulated annealing method via the steepest gradient approach (5000 cycles). Again, molecular dynamics simulations were performed utilizing PME methods to designate long-range electrostatic connections at a cut-off distance of 8 Å at physiological conditions (298 K, pH 7.4, 0.9% NaCl) [155]. Multiple time-step algorithms, combined with a simulation time step interval of 2.50 fs, were selected [156]. Molecular dynamics simulations were executed for 100 ns at consistent pressure, and Berendsen thermostat and MD trajectories were saved every 25 ps; further analysis was performed by default script of YASARA [157] macro and VMD [158] software.

### 3.6. Binding Free Energy Calculation

After the molecular dynamics simulation, MM–PBSA (molecular mechanics–Poisson–Boltzmann surface area) binding free energy calculations were done for all snapshots employing YASARA software using the following formula:

Binding Energy = EpotRecept + EsolvRecept + EpotLigand + EsolvLigand—EpotComplex—EsolvComplex [80,159].

Here, YASARA [158] built-in macros were applied to calculate MM–PBSA binding energy, using AMBER 14 as a force field, where higher positive energies suggest good binding and negative energies do not indicate any binding [79].

### 3.7. Physicochemical, Drug-Likeness, Pharmacokinetic and Toxicokinetic Properties Prediction

Drug ability or drug-likeness, along with the physicochemical properties of *D. orbita* secondary metabolites and aspirin, were predicted using the SwissADME web tools provided by the Swiss Institute of Bioinformatics to determine their physicochemical properties [81]. SwissADME computational filters were also used to assess conformity to Lipinski’s ‘Rule of Five’ (ROF) [160], established by leading pharmaceutical industries and cheminfomaticians to assess the drug-likeness of small molecules. The pharmacological significance of a ligand is also based on its pharmacokinetic and toxicokinetic properties, which are evaluated on the basis of the physicochemical properties of the chemical structure as well as the absorption, distribution, metabolism, excretion, and toxicity (commonly abbreviated as ADMET) properties of the compounds [161]. The ADMET profile of *D. orbita* brominated indole derivatives was performed using the QikProp module executed in the Schrödinger package [147], along with the pkCSM web server (http://structure.bioc.cam.ac.uk/pkcsm, accessed on 1 November 2018). In the last few years, QikProp has been widely recognized as a useful tool for screening potential drug candidates and has proven to be an innovative tool for optimizing the pharmacokinetic profile of pharmaceutically appropriate compounds [162]. Besides the pkCSM server, the most comprehensive and latest manually curated data of various chemicals linked with known ADMET profiles were compared against the query compounds [109]. A combination of both QikProp and pkCSM has been used here for the optimum results.

### 3.8. Distance-Based Linear Modeling of Physicochemical Properties, COX-1 and -2 Binding, Pharmacokinetic and Toxicokinetic Predictions

Distance-based linear models and principal coordinate analysis plots were constructed in PRIMER V7 + PERMANOVA (PRIMER-Auckland, New Zealand). Euclidean distance similarity matrices were constructed on the normalized quantitative biological response variables, which were then correlated to the physicochemical predictor variables using AIC selection criteria and BEST selection procedure, with marginal tests. The models were run using 999 permutations of the data.

## 4. Conclusions

The investigation of the inhibition of proteins with small molecules through in silico screening strategies is of great interest and has come to play a substantial role in drug design and screening. Here, we investigated the docking and molecular dynamics simulation of brominated indoles from the natural marine mollusk *D. orbita* towards COX-1/2, with additional modeling of their physicochemical, drug-likeness, and ADMET properties. Molecular docking score, stability, and compactness within the pocket of the cyclooxygenase enzymes indicated that the *D. orbita* brominated compounds hold promise for the regulation of inflammation, with strong and stable binding predicted through a molecular dynamics simulation study and binding energy calculations. The physicochemical, drug-likeness properties, along with the ADMET study, predict the drug/lead-like potentiality of these natural marine indoles. According to the in silico evaluation, these brominated indole derivatives are predicted to have potential use as novel COX-inhibiting anti-inflammatory agents due to numerous interactions and beneficial properties, as observed in comparison to the standard NSAID compound aspirin. The studies will be useful for directing further in vitro, in vivo, and clinical-based evaluations and for the validation of pharmacokinetic and toxicological properties of brominated indoles from natural marine mollusk *D. orbita* as an anti-inflammatory agent.

## Figures and Tables

**Table 1:** Summary of molecular docking analysis and XP-score results from Schrodinger (Maestro v11.6) for COX-1 (PDB ID: 3N8X), the reference molecule aspirin, and four *Dicathais orbita* compounds.

Ligand Name	XP Docking Score (kcal mol^−1^)	GLIDE Energy (kcal mol^−1^)	GLIDE Model (kcal mol^−1^)	GLIDE Ligand Efficiency
Aspirin	−2.80	−26.25	−33.12	−0.21
Tyrindoxyl sulfate	−6.17	−33.26	−37.64	−0.36
Tyrindoleninone	−6.85	−32.49	−37.17	−0.52
6-Bromoisatin	−6.06	−27.95	−36.96	−0.50
6,6′-Dibromoindirubin	−7.25	−36.23	2.69	−0.33

**Table 2:** Summary of molecular docking analysis and XP-score results from Schrodinger (Maestro v11.6) for COX-2 (PDB ID: 5IKR) for the reference molecule aspirin and four *Dicathais orbita* compounds.

Ligand Name	XP Docking Score (kcal mol^−1^)	GLIDE Energy (kcal mol^−1^)	GLIDE Model (kcal mol^−1^)	GLIDE Ligand Efficiency
Aspirin	−6.87	−31.43	−41.06	−0.52
Tyrindoxyl sulfate	−6.34	−34.58	−44.53	−0.37
Tyrindoleninone	−7.17	−29.27	−30.7	−0.55
6-Bromoisatin	−6.19	−26.1	−32.51	−0.51
6,6′-Dibromoindirubin	−3.14	−15.27	1.96	−0.14

**Table 3:** Physicochemical properties and drug-likeness parameters of secondary metabolites of the *Dicathais orbita* compound in comparison with a standard NSAID.

Parameters	Aspirin	Tyrindoxyl Sulfate	Tyrindoleninone	6-Bromoisatin	6,6′-Dibromoindirubin
IUPAC Name	2-acetyloxybenzoic acid	(6-bromo-2-methylsulfanyl- 1H-indol-3-yl) hydrogen sulfate	6-bromo-2-methylsulfanylindol-3-one	6-bromo-1H-indole-2,3-dione	6-bromo-2-(6-bromo-2-hydroxy-1H-indol-3-yl) indol-3-one
Canonical SMILES	CC(=O)OC1=CC=CC= C1C(=O)O	CSC1=C(C2=C(N1)C= C(C=C2)Br)OS (=O)(=O)O	CSC1=NC2=C(C1=O)C= CC(=C2)Br	C1=CC2=C(C=C1Br) NC(=O)C2=O	C1=CC2=C(C=C1Br)NC(= C2C3=NC4=C(C3=O)C=CC (=C4)Br)O
**Physicochemical properties**					
Molecular formula	C_9_H_8_O_4_	C_9_H_8_BrNO_4_S_2_	C_9_H_6_BrNOS	C_8_H_4_BrNO_2_	C_16_H_8_Br_2_N_2_O_2_
Molecular weight	180.16 g/mol	338.20 g/mol	256.12 g/mol	226.03 g/mol	420.05 g/mol
Fraction Csp3	0.11	0.11	0.11	0.00	0.00
Heavy atoms	13	17	13	12	22
Aromatic heavy atoms	6	9	6	6	15
Molar refractivity (MR)	44.90	69.94	62.35	49.86	96.02
Topological polar surface area (TPSA)	63.60Å²	113.07 Å²	54.73 Å²	46.17 Å²	65.45 Å²
Lipinski violations	0	0	0	0	0
**Lipophilicity**					
iLOGP	1.3-	1.25	2.26	1.14	2.73
XLOGP3	1.19	2.64	2.60	1,33	0.91
MLOGP	1.51	1.52	1.68	0.91	2.95
SILICOS-IT	1.10	1.63	3.69	2.19	5.42
**Water Solubility**					
Log S (ESOL)	−1.85	−3.79	−3.34	−2.45	−5.47
Qualitative solubility	Very soluble	Soluble	Soluble	Soluble	Moderately soluble

**Table 4:** Pharmacokinetic and toxicokinetic (ADMET) properties predicted the profile of secondary metabolites of *Dicathais orbita* compounds compared to the reference molecule by Qikprop and pkCSM.

Parameters	Aspirin	Tyrindoxyl Sulfate	Tyrindoleninone	6-Bromoisatin	6,6′-Dibromoindirubin
**Absorption**					
Human intestinal absorption	76.93%	90.56%	94.99%	92.49%	90.08%
CaCo-2 permeability	0.09	0.94	1.29	1.23	0.54
P-glycoprotein I inhibitor	No	No	No	No	No
P-glycoprotein II inhibitor	No	No	No	No	No
**Distribution**					
Plasma protein binding (QPlogKhsa)	−0.75	−0.41	−0.45	−0.61	0.33
VDss (human)	−1.71	−1.85	0.21	−0.03	0.40
Fraction unbound (human)	0.48	0.49	0.30	0.44	0.04
Blood brain barrier (BBB) permeability	−0.33	−0.77	−0.04	0.36	−0.15
**Metabolism**					
CYP 2D6 Substrate	No	No	No	No	No
CYP 2D6 Inhibitor	No	No	No	No	No
**Excretion**					
Total clearance	0.72	0.17	0.26	0.10	0.23
Renal OCT2 substrate	No	No	No	No	Yes
Toxicity Assays					
AMES toxicity	No	No	No	No	No
Hepato toxicity	No	No	No	No	No
hERG I inhibitor	No	No	No	No	No
Oral rat acute toxicity LD_50_ (mol/kg)	2.28	1.33	2.47	2.42	2.48

**Table 5:** Outcomes of the distance-based linear models for the biological predictions and physicochemical properties of aspirin and the brominated indoles from *Dicathais orbita*. (**A**) *p*-values from the marginal tests for each individual variable. (**B**) Overall BEST solution from the combined models, with a proportion of the variation explained (R^2^).

(A)	Marginal Tests, *p*-Value									
Physico-Chemical Parameter	Cox 1	Cox 2	Intestinal Absorption	CaCo-2 Permeability	QPlog Khsa	VDss	Unbound Fraction	BBB Permeability	Total Clearance	Oral LD50
iLOGP	0.146	0.155	0.716	0.945	0.069	0.193	0.042	0.876	0.986	0.373
XLOGP3	0.056	0.115	0.485	0.895	0.011	0.364	0.103	0.713	0.518	0.989
MLOGP	0.194	0.181	0.945	0.585	0.088	0.596	0.1	0.715	0.923	0.5
SILICOS-IT	0.062	0.065	0.353	0.791	0.04	0.128	0.013	0.667	0.576	0.362
Log S (ESOL)	0.041	0.091	0.419	0.874	0.009	0.417	0.083	0.792	0.409	0.965
Molecular weight	0.049	0.093	0.525	0.943	0.006	0.576	0.149	0.631	0.377	0.876
Fraction Csp3	0.813	0.094	0.885	1	0.415	0.289	0.498	0.273	0.507	0.628
Heavy atoms	0.142	0.125	0.916	0.636	0.03	0.811	0.158	0.526	0.792	0.967
Aromatic heavy atoms	0.149	0.087	0.953	0.728	0.056	0.813	0.209	0.608	0.589	1
Molar refractivity	0.044	0.109	0.464	0.981	0.009	0.476	0.091	0.719	0.495	0.987
Topological polar surface area	0.889	0.558	0.906	0.883	0.833	0.161	0.642	0.02	0.783	0.114
Human Intestinal Absorption				MLOGP, Log S, Molecular weight				0.2		
CaCo-2 Permeability				iLOGP, MLOGP, Molecular weight				1		
Plasma protein binding (QPlogKhsa)				iLOGP, SILICOS-IT, Aromatic heavy atoms				1		
VDss (human)				XLOGP3, Heavy atoms, Aromatic heavy atoms				1		
Unbound fraction (human)				iLOGP, MLOGP, Fraction Csp3				0.1		
Blood-brain barrier (BBB) permeability				iLOGP, Fraction Csp3, Heavy atoms				0.95516		
Total clearance				iLOGP, SILICOS-IT, Fraction Csp3				0.999697		
(**B**)				**BEST model**				**R^2^**		
COX-1				Log S, Molecular refractivity, Total polar surface area				0.98852		
COX-2				iLOGP, Heavy atoms, Aromatic heavy atoms				0.99933		

## Data Availability

Data is contained within the article and Supplementary Material.

## Supplementary Materials

The following are available online, S1-Table S1: Summary of non-bonding interactions analysis for cyclooxygenase-1 (PDB ID: 3N8X), the reference molecule aspirin, and four D. orbita compounds; S2-Table S2: Summary of non-bonding interactions analysis for cyclooxygenase-2 (PDB ID: 5IKR), for the reference molecule aspirin and four D. orbita compounds. S3-Figure S1: Principal coordinates analysis showing the difference in (a) physicochemical properties and (b) pharmacokinetic properties of the brominated indoles from *D. orbita*.
