# Supplementary material for: Mollusc-Derived Brominated Indoles for the Selective Inhibition of Cyclooxygenase: A Computational Expedition
Source: Molecules. 2021 Oct 29;26(21):6538. doi: 10.3390/molecules26216538 (PMC8587571; doi:10.3390/molecules26216538)
Supplement: Supplementary file 1 [file molecules-26-06538-s001.zip › molecules-1422813-supplementary.pdf]

molecules

Supplementary Material

Mollusc Derived Brominated Indoles for the Selective Inhibition of Cyclooxygenase: A Computational Expedition

Md. Mominur Rahman [1], Md. Junaid [2], S. M. Zahid Hosen [2, 3], Mohammad Mostafa [2], Lei Liu [4] and Kirsten Benkendorff [1,5,*]

1 Marine Ecology Research Centre, Faculty of Science and Engineering, Southern Cross University, Lismore, NSW 2480, Australia; m.rahman.23@student.scu.edu.au
2 Molecular Modeling Drug-design and Discovery Laboratory, Pharmacology Research Division, BCSIR Laboratories Chattogram, Bangladesh Council of Scientific and Industrial Research, Chattogram, Bangladesh; md.junaid@northsouth.edu (M.J.); drmostafabcsir@yahoo.com (M.M.)
3 Pancreatic Research Group, South Western Sydney Clinical School, and Ingham Institute for Applied Medical Research, Faculty of Medicine, University of New South Wales, Australia; s.hosen@student.unsw.edu.au
4 Southern Cross Plant Science, Southern Cross University, Lismore, NSW 2480, Australia; ben.liu@scu.edu.au
5 National Marine Science Centre, Southern Cross University, Coffs Harbour, New South Wales, Australia; kirsten.benkendorff@scu.edu.au
* Correspondence: kirsten.benkendorff@scu.edu.au

Table S1. Summary of nonbonding interactions analysis for Cyclooxygenase-1 (PDB ID: 3N8X), for the reference molecule aspirin and four *D. orbita* compounds.

Compounds Name	Hydrogen Bond				Hydrophobic Bond				Electrostatic Bond (EB) / Halogen Bond (HB) / Other Bond (OB)			
	Bonding type	Protein: Interacting amino acids	Ligand: Interacting atoms or ring	Distance	Bonding type	Protein: Interacting amino acids	Ligand: Interacting atoms or ring	Distance	Bonding type	Protein: Interacting amino acids	Ligand: Interacting atoms or ring	Distance
Aspirin	Conventional	Arg-120	H…O	2.04	Pi-Alkyl	Leu-352	X	5.07				
	Conventional	Arg-120	H…O	2.44	Pi-Alkyl	Ile-523	X	4.70				
	Carbon Hydrogen	Ser-530	H…O	2.97	Pi-Alkyl	Ala-527	X	4.49				
Tyrindoxyl Sulfate	Conventional	Arg-120	H…O	2.41	Amide Pistacked	Gly-526	X1	4.47	Attractive Charge	Arg-120	H…O (EB)	2.13
	Carbon Hydrogen	Arg-120	H…O	2.13	Amide Pistacked	Ala-527	X1	4.47				
	Salt Bridge	Ser-353	H…O	2.76	Alkyl	Ile-523	X3	3.83	Pi-Sulfur	His-90	X3 (OB)	5.87
					Pi-Alkyl	Leu-352	X2	5.04				
					Pi-Alkyl	Ile-523	X2	4.56				
					Pi-Alkyl	Ala-527	X2	4.27				
Tyrindoleninone	Carbon Hydrogen	Ile-523	H…O	2.73	Amide Pistacked	Gly-526	X1	4.87	Pi-Sulfur	Met-522	X1 (OB)	5.98
					Amide Pistacked	Ala-527	X4	4.87				
					Amide Pistacked	Gly-526	X4	3.96				
					Amide Pistacked	Ala-527	X1	3.96				
					Alkyl	Ile-523	X3	4.77				
					Pi-Alkyl	Tyr-355	X3	4.19				
					Pi-Alkyl	Val-349	X4	5.21				
					Pi-Alkyl	Leu-352	X4	4.82				
					Pi-Alkyl	Ala-527	X4	3.93				
					Pi-Alkyl	Leu-352	X1	5.18				
					Pi-Alkyl	Ala-527	X1	4.71				
6-Bromoisatin					Amide Pi-stacked	Gly-526	X5	3.88				
					Amide Pi-stacked	Ala-527	X1	3.88				
					Amide Pi-stacked	Gly-526	X1	4.97				
					Amide Pi-stacked	Ala-527	X5	4.97				
					Pi-Alkyl	Leu-352	X5	5.17				
					Pi-Alkyl	Ala-527	X5	4.38				
					Pi-Alkyl	Val-349	X1	5.05				
					Pi-Alkyl	Leu-352	X1	5.04				
					Pi-Alkyl	Ala-527	X1	3.71				
6,6′ dibromoindirubin	Carbon Hydrogen	Ser-353	H…O	2.59	Amide Pi-stacked	Gly-526	X6	4.83	Attractive charge	Arg-120	N-H…O (EB)	5.41
					Amide Pi-stacked	Ala-527	X1	4.83				
					Amide Pi-stacked	Gly-526	X1	3.96				
					Amide Pi-stacked	Ala-527	X6	3.96				
	Pi-donor Hydrogen	Tyr-355	H…O	2.59	Alkyl	Leu-384	Br	4.86	Pi-Anion	Tyr-355	O (EB)	4.20
					Pi-Alkyl	Phe-381	Br					
					Pi-Alkyl	Tyr-385	Br					
					Pi-Alkyl	Trp-387	Br					
					Pi-Alkyl	Val-349	X6					
					Pi-Alkyl	Ala-527	X6					
6,6′ dibromoindirubin (Continue)					Pi-Alkyl	Val-349	X6					
					Pi-Alkyl	Leu-352	X6					
					Pi-Alkyl	Ile-523	X6					
					Pi-Alkyl	Ala-527	X6					
					Pi-Alkyl	Val-349	X1					
					Pi-Alkyl	Leu-359	X1					
					Pi-Alkyl	Ala-527	X1					
					Pi-Alkyl	Leu-531	X1					
					Pi-Alkyl	Leu-352	X1					

Ala-527	X1

Here, X, X1, X2, X3 indicates that, X = Benzene, X1 = 6-Bromo benzene, X2 =Indole-3-ol, X3 = (Methylthio)methane, X4 = Indole-3-one, X5 = Indoline-2,3-dione, X6 = Indolin-2-one.

Table S2. Summary of nonbonding interactions analysis for Cyclooxygenase-2 (PDB ID: 5IKR), for the reference molecule aspirin and four *D. orbita* compounds.

Compounds Name	Hydrogen Bond				Hydrophobic Bond				Electrostatic Bond (EB) / Halogen Bond (HB) / Other Bond (OB)			
	Bonding Type	Protein: Interacting Amino Acids	Ligand: Interacting Atoms or Rings	Distance	Bonding Type	Protein: Interacting Amino Acids	Ligand: Interacting Atoms or Rings	Distance	Bonding Type	Protein: Interacting Amino Acids	Ligand: Interacting Atoms or Rings	Distance
Aspirin	Conventional Hydrogen	Tyr-385	H…O	1.79	Amide Pi-stacked	Gly-526	X	3.76				
		Ser-530	H…O	1.83		Ala-527	X	3.76				
					Pi-Alkyl	Leu-352	X	5.13				
						Ala-527	X	4.82				
Tyrindoxyl Sulfate	Conventional Hydrogen	Tyr-385	H…O	2.11	Pi-Alkyl	Val-349	X2	5.03	Pi-Sulfur	Trp-387	X3 (OB)	5.72
		Ser-530	H…O	2.22		Leu-352	X2	4.85		Trp-387	O (OB)	5.83
	Carbon Hydrogen	Ser-530	H…O	2.82		Val-523	X1	5.38				
		Ser-530	H…O	2.50								
		Ser-530	H…O	2.93		Ala-527	X1	3.57				
						Val-349	X	4.25				
						Ala-527	X	3.47				
						Leu-531	X	4.12				
Tyrindolenenone	Conventional Hydrogen	Ser-530	H…O	2.10	Amide Pi-stacked Alkyl	Gly-526	X1	4.12	Halogen	Met-522	Br	3.12
						Ala-527	X4	4.12				
						Gly-526	X4	3.80				
						Ala-527	X1	3.80				
	Hydrogen	Ser-530	H…O	2.71	Alkyl	Ala-527	X3	3.63				
						Val-349	X3	4.39				
						Leu-531	X3	3.72				
					Pi-Alkyl	Val-349	X4	5.15				
						Leu-352	X4	4.83				
						Ala-527	X4	3.92				
						Leu-352	X1	5.17				
						Ala-527	X1	5.14				
6-Bromoisatin	Conventional Hydrogen	Ser-530	H…O	2.12	Amide Pi-stacked	Gly-526	X1	4.12	Halogen	Met-522	Br	3.13
						Ala-527	X4	4.12				
						Gly-526	X4	3.80				
						Ala-527	X1	3.80				
	Carbon Hydrogen	Ser-530	H…O	2.70	Pi-Alkyl	Val-349	X5	5.10				
						Leu-352	X5	4.78				
						Ala-527	X5	3.92				
						Leu-352	X1	5.14				
						Ala-527	X1	5.10				
6,6′dibromoindirubin	Carbon Hydrogen	Ser-530	H…O	2.92	Pi-pi T shaped	Tyr-355	X1	4.74				
	Pi-donor Hydrogen	Arg-120	H…O	3.00	Amide pi-stacked	Gly-526	X6	4.95				
						Ala-527	X6	4.95				
						Gly-526	X1	4.06				
						Ala-527	X1	4.06				
					Alkyl	Leu-352	Br	5.09				
						Met-522	Br	4.95				
					Pi-Alkyl	Trp-387	Br	5.22				
						Phe-518	Br	4.46				
						Val-116	X6	4.86				

		Val-349	X6	5.35
		Leu-359	X6	4.98
		Ala-527	X6	4.93
		Leu-531	X6	4.71
		Val-349	X6	4.32
		Leu-352	X1	4.81
		Ala-527	X1	3.97

Here, X, X1, X2, X3 indicates that, X = Benzene, X1 = 6-Bromo benzene, X2 =Indole-3-ol, X3 = (Methylthio)methane, X4 = Indole-3-one, X5 = Indoline-2,3-dione, X6 = Indolin-2-one.

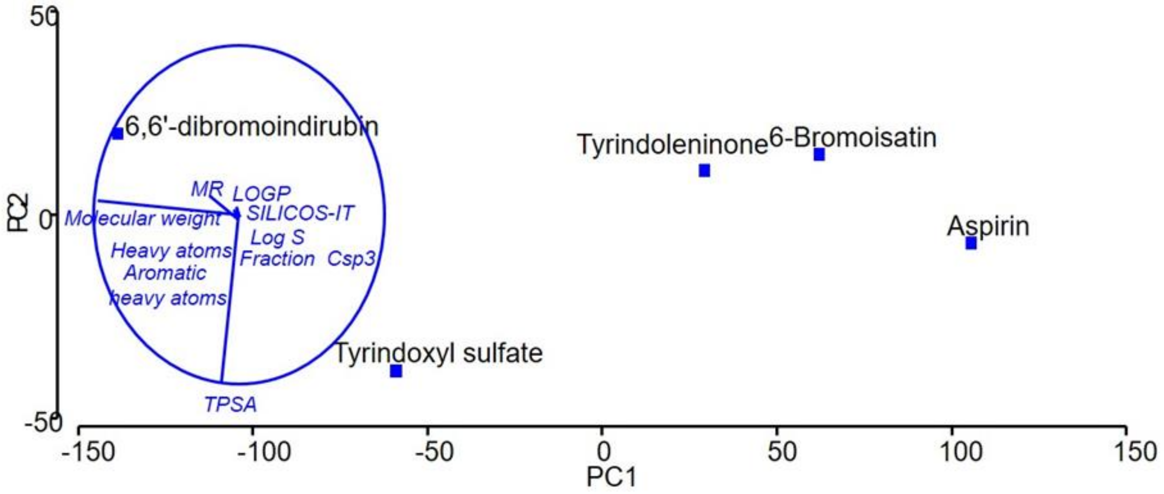

a)

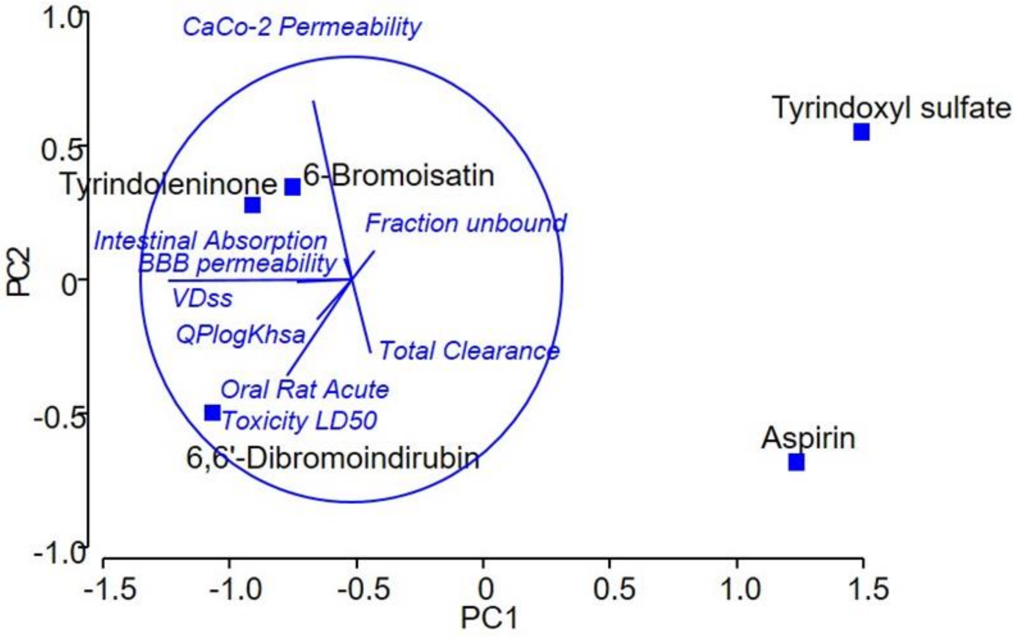

b)

Figure S1. Principle coordinate ordination showing the difference in **a**) physicochemical properties and **b**) pharmacokinetic properties of the brominated indoles from *Dicathais orbita.*
